# Molecular Characterization of African Swine Fever Virus From 2019-2020 Outbreaks in Guangxi Province, Southern China

**DOI:** 10.3389/fvets.2022.912224

**Published:** 2022-06-15

**Authors:** Kaichuang Shi, Huixin Liu, Yanwen Yin, Hongbin Si, Feng Long, Shuping Feng

**Affiliations:** ^1^Guangxi Center for Animal Disease Control and Prevention, Nanning, China; ^2^College of Animal Science and Technology, Guangxi University, Nanning, China

**Keywords:** African swine fever virus, *B646L (p72)* gene, *E183L (p54)* gene, *EP402R (CD2v)* gene, genotype, serogroup, phylogenetic analysis

## Abstract

African swine fever virus (ASFV) causes contagious hemorrhagic disease of pigs with high morbidity and mortality. To identify the molecular characteristics of ASFV strains circulating in Guangxi province, southern China, a total of 336 tissue samples collected from 336 domestic pigs that died as a result of severe hemorrhagic disease during 2019–2020 were tested for ASFV. Furthermore, 66 ASFV strains were genetically characterized by sequence analysis of the C-terminal region of *B646L* (*p72*) gene, the complete *E183L* (*p54*) gene, the variable region of *EP402R* (*CD2v*) gene, the central variable region (CVR) of *B602L* gene, the full *MGF505-2R* gene, and the tandem repeat sequence (TRS) within intergenic region (IGR) between the *I73R* and *I329L* (*I73R/I329L*) genes. Phylogenetic analysis revealed that the ASFV strains from Guangxi province belonged to genotypes I and II based on the *B646L* (*p72*) and *E183L* (*p54*) genes, and there were eight different tetrameric TRS variants based on the CVR of *B602L* gene. Phylogenetic analysis of the *EP402R* (*CD2v*) gene revealed that these ASFV strains belonged to serogroups 4 and 8. Eight of the 66 strains belonged to genotype I and serogroup 4, and showed deletion of whole *MGF505-2R* gene. The sequence analysis of the IGR between the *I73R/I329L* genes showed that IGR II and III variants were co-circulating in Guangxi province. The results indicated that ASFV strains circulating in Guangxi province during 2019–2020 outbreaks showed high genetic diversity, of which genotypes I and II, as well as serogroups 4 and 8, were simultaneously circulating in Guangxi province, and there existed wild-type and naturally gene-deleted strains in the field. This is the first detailed report on the molecular characterization of the ASFV strains circulating in southern China, and serogroup 4 in China.

## Introduction

African swine fever virus (ASFV) is an enveloped double-stranded DNA virus of the genus *Asfivirus* in the family *Asfarviridae* (1). ASFV causes African swine fever (ASF), a devastating hemorrhagic disease infecting domestic pigs and wild boars, with high mortality rate up to 100% (2). ASF was first identified in Kenya in the 1920s, Europe in 1957, the Caucasus region and southern Russia in 2007 (3, 4), China in August 2018 (5), and other Asian countries, such as Mongolia, Korea, Vietnam, Laos, Cambodia, the Philippines, and Indonesia, since the end of 2018 (6, 7). Currently, the outbreaks of ASF are still ongoing in Africa, the trans-Caucasus region, eastern Europe, Russian Federation, Asia, and Latin America, which pose a huge challenge to the swine industry in these regions (8, 9). ASF has caused huge economic losses to the swine industry worldwide since the 1920s, and is a notifiable disease to the World Organization for Animal Health (OIE).

The ASFV genome varies by ~170–193 kb and encodes over 168 kinds of protein (10). Based on the partial *B646L* (*p72*) gene sequences, ASFV strains from different countries are currently classified into 24 genotypes (11, 12). Of all the 24 genotypes of ASFV found in Africa, only genotypes I and II have outbroken outside Africa until now (13, 14). Genotype I first occurred in Portugal in 1957, and then in other European countries, southern America, and Caribbean island countries (15). Genotype II entered Georgia from southern Africa in 2007 (16), later spread to eastern Europe and westward into western Europe arriving in Belgium in September 2018 (17), and introduced into Asia arriving in China in August 2018 (18). Since then, genotype II strains have spiraled out of control, spreading to multiple Chinese provinces and reaching other Asian countries, such as Vietnam, Cambodia, Laos, and Korea (8, 9).

Since China raises more than 50% of the world's pigs every year, it is very important to master the molecular characterization of ASFV strains circulating in China. ASF was first identified in China in August 2018 (5), where it rapidly spread to most provinces in China within a short time (19). To date, genotypes I and II ASFV strains have been reported in China (20, 21), and the wild-type and gene-deleted ASFV strains have been identified in several provinces in China (22, 23). However, the molecular characterization of ASFV strains circulating in China has been limited, thus the ASFV genotypes mapping in the country is incomplete until now. In this study, the tissue samples, which were collected in the field during the ASF outbreaks from 2019 to 2020 in Guangxi province, southern China, were tested for ASFV by real-time quantitative polymerase chain reaction (qPCR). The positive samples were randomly selected to amplify and sequence the C-terminal region of *B646L* gene encoding protein 72 (p72), the complete *E183L* gene encoding protein 54 (p54), the central variable region (CVR) of *B602L* gene, the full *MGF505-2R* gene, and the tandem repeat sequence (TRS) of intergenic region (IGR) between the *I73R* and *I329L* genes (*I73R/I329L* genes), and analyze the genetic characteristics and variations of ASFV strains circulating in Guangxi province. Furthermore, the serogroups of ASFV strains were determined by sequence analysis of the variable region of *EP402R* gene encoding protein CD2v (pCD2v). This is the first detailed report on the molecular characterization of the ASFV strains circulating in southern China.

## Materials and Methods

### Sample Collection and Detection of ASFV Genome

From January 2019 to December 2020, a total of 336 tissue samples from 336 domestic pigs were collected from 86 different pig farms in 14 regions of Guangxi province, southern China in this study. The pig farms with high fever, hemorrhagic pig herds suspected of ASF were selected, and three to five dead pigs form each farm were selected randomly to collect clinical samples. The tissue samples, including lung, liver, spleen and lymph nodes from each dead pig, were collected, transported immediately at ≤4°C to the laboratory, and stored at −70°C until used. The tissue samples were homogenized (10%, W/V) in phosphate-buffered saline (PBS, pH7.2) using a blender (Tianlong, Xi'an, China), followed by shaking with small glass beads for 5 min, and then freeze-thawed three times, centrifuged at 12, 000 × g at 4°C for 5 min. Total DNA was extracted from the supernatants using MiniBEST RNA/DNA Extraction Kit (TaKaRa, Beijing, China) and confirmed the presence of ASFV by a real-time qPCR targeting the *B646L* (*p72*) gene by the following specific primers and probe: ASFV-U: GGCGTATAAAAAGTCCAGGAAATTC, ASFV-D: TTCGGCGAGCGCTTTATC, ASFV-P: Texas Red-TCACCAAATCCTTTTGCGATGCAAGCT-BHQ2. The parameters of real-time qPCR were as follows: 10 μl of Premix Ex Taq (TaKaRa, Beijing, China), 0.4 μl of each of ASFV primers (20 pmol/μl), 0.5 μl of ASFV probe (20 pmol/μl), 2 μl of total DNA as templates and distilled water to a total volume of 20 μl. The amplification parameters were as follows: 95°C for 1 min; and then 40 cycles of 95°C for 5 s, 59°C for 34 s. The fluorescent signals were determined at the end of each cycle. The ASFV positive samples were randomly selected for genetic analysis.

### Amplification and Sequencing of the Targeted Genes

The positive samples were randomly selected for amplifying the C-terminal region of the *B646L* (*p72*) gene, the *E183L* (*p54*) gene, the CVR of the *B602L* gene, the *EP402R* (*CD2v*) gene, the full *MGF505-2R* gene, and the TRS within IGR between the *I73R/I329L* genes, using six different pairs of primers (Table 1) according to the previous reports (24–29) with some modifications. The targeted genes were amplified by PCR using the Tks Gflex™ DNA Polymerase kit (TaKaRa, Beijing, China), and the products were electrophoresed in a 1.2% agarose gel and visualized using an imaging system (UVITEC, France). The amplified fragments were purified and cloned into a pMD18-T vector (TaKaRa, Beijing, China), transferred into *E. coli* DH5α competent cells (TaKaRa, Beijing, China) and sequenced with an ABI 3730XL sequencer (ABI, Los Angeles, CA, United States).

**Table 1:** Primers used for evaluation and amplification of ASFV in this study.

**Target**	**Name**	**Primer sequence (5^′^-3^′^)**	**References**
*B646L* (*p72*)	p72-U	GGCACAAGTTCGGACATGT	(24)
	p72-D	GTACTGTAACGCAGCACAG	
*E183L* (*p54*)	p54-U	CGAAGTGCATGTAATAAACGTC	(25)
	p54-D	TGTAATTTCATTGCGCCACAAC	
*B602L* (CVR)	B602L-U	AATGCGCTCAGGATCTGTTAAATCGG	(26)
	B602L-D	TCTTCATGCTCAAAGTGCGTATACCT	
*MGF505-2R*	MGF505-2R-U	GCAGAGGTATGATGTCCTTA	(27)
	MGF505-2R-D	TTCCTGTTGAACAAGTATCT	
*I73R/I329L*	I73R/I329L-U	CCATTTATCCCCCGCTTTGG	(28)
	I73R/I329L-D	TCGTCATCCTGAGACAGCAG	
*EP402R* (*CD2v*)	CD2v-U	YCTGTTGATTCCCCAACTATTACA	(29)
	CD2v-D	ATGGCGGGATATTGGGTAGT	

### Genomic Analysis

Nucleotide and amino acid alignments were carried out using the EditSeq and Megalign program in the DNAstar package (DNAstar, Madison, WI, United States). All the reference sequences of ASFV were downloaded from the GenBank in the National Center for Biotechnology Information (NCBI) (Supplementary Tables S1–S3). Phylogenetic analysis based on the *B646L* (*p72*) gene and the *E183L* (*p54*) gene nucleotide sequences, respectively, were conducted using MEGA X, and the genotypes of ASFV strains were determined as previously described (30–32). The serogroups of the obtained ASFV strains were achieved by phylogenetic analysis of the *EP402R* (*CD2v*) gene sequences using MEGA X as previously described (33, 34). The maximum likelihood (ML) and the best-fit model methods were used to compare the differences between the two typing methods and analyze gene sequences. Phylogeny was inferred following 1,000 bootstrap replications.

The nucleotides of the CVR of the *B602L* gene and the TRS within the IGR between *I73R/I329L* genes were first translated into amino acids and searched by similarity analysis according to the ASFV amino acid sequences using BLASTp (v2.2.2.29). The CVR amino acid tetramers were matched with those previously reported codes (31, 34, 35).

## Results

### Detection of ASFV Genome in Clinical Samples

Of all 336 clinical samples evaluated by real-time qPCR using primers ASFV-U/D and probe ASFV-P, 192 samples were positive for ASFV, with a positive rate of 57.14% (192/336). Sixty-six samples were randomly selected from these 192 positive samples and further confirmed by real-time qPCR using a commercial ASFV qPCR detection kit (Lijian, China) which targeted the *B646L* (*p72*), *EP402R* (*CD2v*) and *MGF505-2R* genes, before they were used for gene amplification, sequencing and analysis. The results showed that all the 66 samples were positive for ASFV based on the *B646L* (*p72*) and *EP402R* (*CD2v*) genes, of which eight samples lacked *MGF505-2R* gene. Then, these samples were further amplified and sequenced to verify the deletion of *MGF505-2R* gene.

### Amplification of the Targeted Genes of ASFV

A total of 66 positive samples were randomly selected for amplifying six targeted genes, including *B646L* (*p72*), *E183L* (*p54*), *EP402R* (*CD2v*), *B602L* (CVR), *MGF505-2R* and IGR between *I73R/I329L* genes. The results showed that the *B646L* (*p72*), *E183L* (*p54*), *EP402R* (*CD2v*), *B602L* (CVR), and IGR between *I73R/I329L* genes were amplified from all the 66 positive samples. However, the *MGF505-2R* gene fragments could be amplified from 58 of the 66 samples. The targeted fragments were purified, cloned and sequenced. The generated sequences of the 66 strains from Guangxi province have been deposited in GenBank under accession numbers OM986132-OM986197 for the *B646L* (*p72)* gene, OM986396-OM986461 for the *E183L* (*p54*) gene, OM986264-OM986329 for the *EP402R* (*CD2v*) gene, OM986198-OM986263 for the *B602L* (CVR) gene, OM986330-OM986395 for the IGR between *I73R/I329L* genes, and OM986074-OM986131 for the *MGF505-2R* gene (Table 2).

**Table 2:** Summary of the 66 ASFV strains from domestic pigs in Guangxi province in this study.

**No**.	**Strain**	**Date**	***B646L***	***E183L***	**CVR profile**	**TRS**	***ER402R***	**GenBank accession number**					
								***B646L* (*p72*)**	***E183L* (*p54*)**	***EP402R* (*CD2v*)**	***B602L* (CVR)**	**IGR between *I73R/I329L***	***MGF505-2R***
1	China/GX/201901	Jan-2019	II	IIa	BNDBNDBNAA	Tet-10a	8	OM986132	OM986396	OM986264	OM986198	OM986330	OM986074
2	China/GX/201902	Jan-2019	II	IIa	BNDBNDBNAA	Tet-10a	8	OM986133	OM986397	OM986265	OM986199	OM986331	OM986075
3	China/GX/201903	Jan-2019	II	IIa	BNDBNDBNAA	Tet-10a	8	OM986134	OM986398	OM986266	OM986200	OM986332	OM986076
4	China/GX/201904	Mar-2019	II	IIa	BNDBND-NAA	Tet-9a	8	OM986135	OM986399	OM986267	OM986201	OM986333	OM986077
5	China/GX/201905	May-2019	II	IIa	BNDBNDBNAA	Tet-10a	8	OM986136	OM986400	OM986268	OM986202	OM986334	OM986078
6	China/GX/201906	May-2019	II	IIa	—–(NK)—–	(NK)	8	OM986137	OM986401	OM986269	OM986203	OM986335	OM986079
7	China/GX/201907	May-2019	II	IIa	BNDBNDBNAA	Tet-10a	8	OM986138	OM986402	OM986270	OM986204	OM986336	OM986080
8	China/GX/201908	May-2019	II	IIa	BNDBNDBNAA	Tet-10a	8	OM986139	OM986403	OM986271	OM986205	OM986337	OM986081
9	China/GX/201909	May-2019	II	IIa	BNDBNDBNAA	Tet-10a	8	OM986140	OM986404	OM986272	OM986206	OM986338	OM986082
10	China/GX/201910	May-2019	II	IIa	BNDBNDBNAA	Tet-10a	8	OM986141	OM986405	OM986273	OM986207	OM986339	OM986083
11	China/GX/201911	May-2019	II	IIa	BNDBNDBNAA	Tet-10a	8	OM986142	OM986406	OM986274	OM986208	OM986340	OM986084
12	China/GX/201912	Aug-2019	II	IIa	BNDBN—AA	Tet-7	8	OM986143	OM986407	OM986275	OM986209	OM986341	OM986085
13	China/GX/201913	Aug-2019	II	IIa	BNDBNDBNAA	Tet-10a	8	OM986144	OM986408	OM986276	OM986210	OM986342	OM986086
14	China/GX/201914	Aug-2019	II	IIa	BNDBNDBNAA	Tet-10a	8	OM986145	OM986409	OM986277	OM986211	OM986343	OM986087
15	China/GX/201915	Aug-2019	II	IIa	BNDBNDBNAA	Tet-10a	8	OM986146	OM986410	OM986278	OM986212	OM986344	OM986088
16	China/GX/201916	Aug-2019	II	IIa	BNDBNDBNAA	Tet-10a	8	OM986147	OM986411	OM986279	OM986213	OM986345	OM986089
17	China/GX/201917	Aug-2019	II	IIa	BNDBNDBNAA	Tet-10a	8	OM986148	OM986412	OM986280	OM986214	OM986346	OM986090
18	China/GX/201918	Aug-2019	II	IIa	BNDBNDBNAA	Tet-10a	8	OM986149	OM986413	OM986281	OM986215	OM986347	OM986091
19	China/GX/201919	Aug-2019	II	IIa	BNDBNDBNAA	Tet-10a	8	OM986150	OM986414	OM986282	OM986216	OM986348	OM986092
20	China/GX/201920	Aug-2019	II	IIa	BNDBNDBNAA	Tet-10a	8	OM986151	OM986415	OM986283	OM986217	OM986349	OM986093
21	China/GX/201921	Sep-2019	II	IIa	BNDBNDBNAA	Tet-10a	8	OM986152	OM986416	OM986284	OM986218	OM986350	OM986094
22	China/GX/201922	Sep-2019	II	IIa	BNDBNDBNAA	Tet-10a	8	OM986153	OM986417	OM986285	OM986219	OM986351	OM986095
23	China/GX/201923	Sep-2019	II	IIa	BNDBNDBNAA	Tet-10a	8	OM986154	OM986418	OM986286	OM986220	OM986352	OM986096
24	China/GX/201924	Sep-2019	II	IIa	BNDBNDBNAA	Tet-10a	8	OM986155	OM986419	OM986287	OM986221	OM986353	OM986097
25	China/GX/201925	Sep-2019	II	IIa	BNDBNDBNAA	Tet-10a	8	OM986156	OM986420	OM986288	OM986222	OM986354	OM986098
26	China/GX/201926	Sep-2019	II	IIa	BNDBNDBNAA	Tet-10a	8	OM986157	OM986421	OM986289	OM986223	OM986355	OM986099
27	China/GX/201927	Oct-2019	II	IIa	BNDBNDBNAA	Tet-10a	8	OM986158	OM986422	OM986290	OM986224	OM986356	OM986100
28	China/GX/201928	Oct-2019	II	IIa	BNDBNDBNAA	Tet-10a	8	OM986159	OM986423	OM986291	OM986225	OM986357	OM986101
29	China/GX/201929	Oct-2019	II	IIa	BNDBNDBNAA	Tet-10a	8	OM986160	OM986424	OM986292	OM986226	OM986358	OM986102
30	China/GX/201930	Oct-2019	II	IIa	BNDBNDBNAA	Tet-10a	8	OM986161	OM986425	OM986293	OM986227	OM986359	OM986103
31	China/GX/201931	Oct-2019	II	IIa	BNDBNDBNAA	Tet-10a	8	OM986162	OM986426	OM986294	OM986228	OM986360	OM986104
32	China/GX/201932	Oct-2019	II	IIa	BNDBNDBNAA	Tet-10a	8	OM986163	OM986427	OM986295	OM986229	OM986361	OM986105
33	China/GX/201933	Oct-2019	II	IIa	BND-NDBNAA	Tet-9b	8	OM986164	OM986428	OM986296	OM986230	OM986362	OM986106
34	China/GX/201934	Oct-2019	II	IIa	BNDBNDBNAA	Tet-10a	8	OM986165	OM986429	OM986297	OM986231	OM986363	OM986107
35	China/GX/201935	Oct-2019	II	IIa	BNDBNDBNAA	Tet-10a	8	OM986166	OM986430	OM986298	OM986232	OM986364	OM986108
36	China/GX/201936	Oct-2019	II	IIa	BNDBNDBNAA	Tet-10a	8	OM986167	OM986431	OM986299	OM986233	OM986365	OM986109
37	China/GX/201937	Oct-2019	II	IIa	BNDBNDBNAA	Tet-10a	8	OM986168	OM986432	OM986300	OM986234	OM986366	OM986110
38	China/GX/201938	Oct-2019	II	IIa	BNDBNDFNAA	Tet-10b	8	OM986169	OM986433	OM986301	OM986235	OM986367	OM986111
39	China/GX/201939	Oct-2019	II	IIa	BNDBNDBNAA	Tet-10a	8	OM986170	OM986434	OM986302	OM986236	OM986368	OM986112
40	China/GX/201940	Oct-2019	II	IIa	BNDBNDBNAA	Tet-10a	8	OM986171	OM986435	OM986303	OM986237	OM986369	OM986113
41	China/GX/201941	Oct-2019	II	IIa	BNDBNDBNAA	Tet-10a	8	OM986172	OM986436	OM986304	OM986238	OM986370	OM986114
42	China/GX/201942	Oct-2019	II	IIa	BNDBNDBNAA	Tet-10a	8	OM986173	OM986437	OM986305	OM986239	OM986371	OM986115
43	China/GX/201943	Oct-2019	II	IIa	BNDBNDBNAA	Tet-10a	8	OM986174	OM986438	OM986306	OM986240	OM986372	OM986116
44	China/GX/201944	Oct-2019	II	IIa	BNDBNDBNAA	Tet-10a	8	OM986175	OM986439	OM986307	OM986241	OM986373	OM986117
45	China/GX/201945	Oct-2019	II	IIa	BNDBNDBNAA	Tet-10a	8	OM986176	OM986440	OM986308	OM986242	OM986374	OM986118
46	China/GX/201946	Oct-2019	II	IIa	BNDBNDBNAA	Tet-10a	8	OM986177	OM986441	OM986309	OM986243	OM986375	OM986119
47	China/GX/201947	Oct-2019	II	IIa	BNDBNDBNAA	Tet-10a	8	OM986178	OM986442	OM986310	OM986244	OM986376	OM986120
48	China/GX/201948	Nov-2019	II	IIa	BNDBNDBNAA	Tet-10a	8	OM986179	OM986443	OM986311	OM986245	OM986377	OM986121
49	China/GX/201949	Nov-2019	II	IIa	BNDBNDBNAA	Tet-10a	8	OM986180	OM986444	OM986312	OM986246	OM986378	OM986122
50	China/GX/201950	Nov-2019	II	IIa	BNDBNDBNAA	Tet-10a	8	OM986181	OM986445	OM986313	OM986247	OM986379	OM986123
51	China/GX/202001	Jan-2020	II	IIa	BNDBNDBNAA	Tet-10a	8	OM986182	OM986446	OM986314	OM986248	OM986380	OM986124
52	China/GX/202002	Jan-2020	I	Ia	BN——AA	Tet-4	4	OM986183	OM986447	OM986315	OM986249	OM986381	/
53	China/GX/202003	Jan-2020	I	Ia	BNDBNDBNAA	Tet-10a	4	OM986184	OM986448	OM986316	OM986250	OM986382	/
54	China/GX/202004	Jan-2020	I	Ia	BNDBNDBNAA	Tet-10a	4	OM986185	OM986449	OM986317	OM986251	OM986383	/
55	China/GX/202005	Jan-2020	I	Ia	BNDBNDBNAA	Tet-10a	4	OM986186	OM986450	OM986318	OM986252	OM986384	/
56	China/GX/202006	Jan-2020	II	IIa	BNDBNDBNAA	Tet-10a	8	OM986187	OM986451	OM986319	OM986253	OM986385	OM986125
57	China/GX/202007	Jan-2020	I	Ia	BNDBNDBNAA	Tet-10a	4	OM986188	OM986452	OM986320	OM986254	OM986386	/
58	China/GX/202008	Jan-2020	I	Ia	BNDBNDBNBA	Tet-10c	4	OM986189	OM986453	OM986321	OM986255	OM986387	/
59	China/GX/202009	Jan-2020	I	Ia	BNDBNDBNAA	Tet-10a	4	OM986190	OM986454	OM986322	OM986256	OM986388	/
60	China/GX/202010	Jan-2020	I	Ia	BNDBNDBNAA	Tet-10a	4	OM986191	OM986455	OM986323	OM986257	OM986389	/
61	China/GX/202011	Feb-2020	II	IIa	BNDBNDBNAA	Tet-10a	8	OM986192	OM986456	OM986324	OM986258	OM986390	OM986126
62	China/GX/202012	Sep-2020	II	IIa	BNDBNDBNAA	Tet-10a	8	OM986193	OM986457	OM986325	OM986259	OM986391	OM986127
63	China/GX/202013	Nov-2020	II	IIa	BNDBNDBNAA	Tet-10a	8	OM986194	OM986458	OM986326	OM986260	OM986392	OM986128
64	China/GX/202014	Nov-2020	II	IIa	BNDBNDBNAA	Tet-10a	8	OM986195	OM986459	OM986327	OM986261	OM986393	OM986129
65	China/GX/202015	Dec-2020	II	IIa	BNDBNDBNAA	Tet-10a	8	OM986196	OM986460	OM986328	OM986262	OM986394	OM986130
66	China/GX/202016	Dec-2020	II	IIa	BNDBNDBNAA	Tet-10a	8	OM986197	OM986461	OM986329	OM986263	OM986395	OM986131

### Sequence Alignment of the *MGF505-2R* Gene

**Figure 1:**
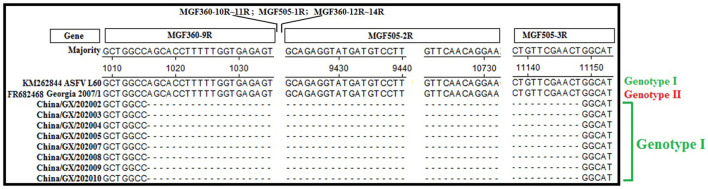
Nucleotide sequence alignment of the strains lacking the *MGF505-2R* gene. Eight clinical samples lacking the *MGF505-2R* gene by the commercial qPCR detection kit (Lijian, China) were further verified by sequence alignment, and confirmed to be lacking the partial *MGF360-9R*, whole *MGF360-10R*~*MGF360-14R, MGF505-1R* and *MGF505-2R*, and partial *MGF505-3R* genes with a total deletion of 10,134 bp.

**Figure 2:**
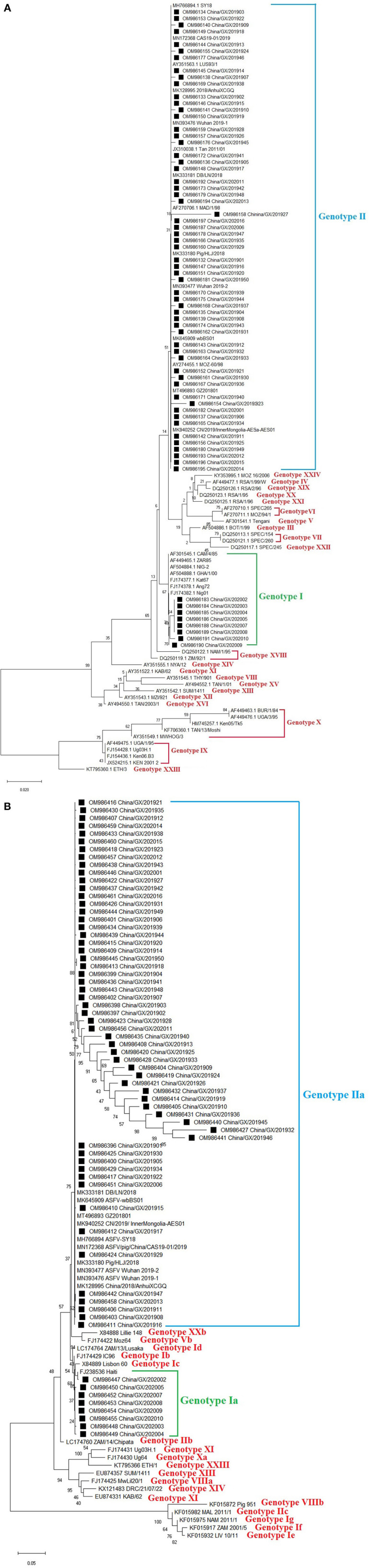
Phylogenetic trees based on nucleotide sequences of the *B646L* (*p72*) **(A)** and *E183L* (*p54*) **(B)** genes. The evolutionary history was inferred by the maximum likelihood method based on the Kimura 2-parameter model [*B646L* (*p72*) gene] and the Kimura 2-parameter + G model [*E183L* (*p54*) gene]. The phylogeny was inferred following 1,000 bootstrap replications, and node values showed percentage bootstrap support. **(A)** Phylogenetic analysis of the *B646L* (*p72*) gene. The ASFV strains from Guangxi province were clustered into genotypes I and II. **(B)** Phylogenetic analysis of the *E183L* (*p54*) gene. The ASFV strains from Guangxi province were clustered into genotypes Ia and IIa. The strains from this study were highlighted with black squares (■) before the names.

The evaluation of ASFV positive samples using a commercial qPCR detection kit (Lijian, China) suggested that eight samples lacked *MGF505-2R* gene. To verify this result, the *MGF505-2R* genes of all the 66 samples were amplified, sequenced and aligned. The results confirmed the deletion of the *MGF505-2R* gene in eight samples (Figure 1). The further analysis showed that these 8 strains belonged to genotype I based on the phylogenetic analysis of the *B646L* (*p72*) gene (Figure 2A) and serogroup 4 based on the phylogenetic analysis of the *EP402R* (*CD2v*) gene (Figure 2B).

### Phylogenetic Analysis of the *B646L* (*p72*) Gene

The *B646L* (*p72*) gene was amplified, sequenced and analyzed. Phylogenetic analysis of *B646L* (*p72*) nucleotide sequences revealed that all ASFV strains, including the 66 strains obtained in this study (Table 2) and the reference strains downloaded from GenBank (Supplementary Table 1), could be clustered into 24 genotypes. The 66 strains from this study distributed in genotypes I and II, of which 8 strains belonged to genotype I and 58 strains belonged to genotype II (Figure 2A).

### Phylogenetic Analysis of the *E183L* (*p54*) Gene

The *E183L* (*p54*) gene was amplified, sequenced and analyzed. Since all the 66 strains from Guangxi province belonged to genotypes I and II based on phylogenetic analysis of the *B646L* (*p72*) gene, we wanted to know whether these strains also belonged to genotypes I and II based on phylogenetic analysis of the *E183L* (*p54*) gene. The result revealed that all the 66 strains from this study (Table 2) and the reference strains downloaded from GenBank (Supplementary Table 2) could be clustered into different genotypes, and further into different sub-genotypes. Of all the 66 strains from this study, 8 strains belonged to sub-genotype Ia, and the other 58 strains belonged to sub-genotype IIa which could be further divided into different branches (Figure 2B).

### Phylogenetic Analysis of the *EP402R* (*CD2v*) Gene

**Figure 3:**
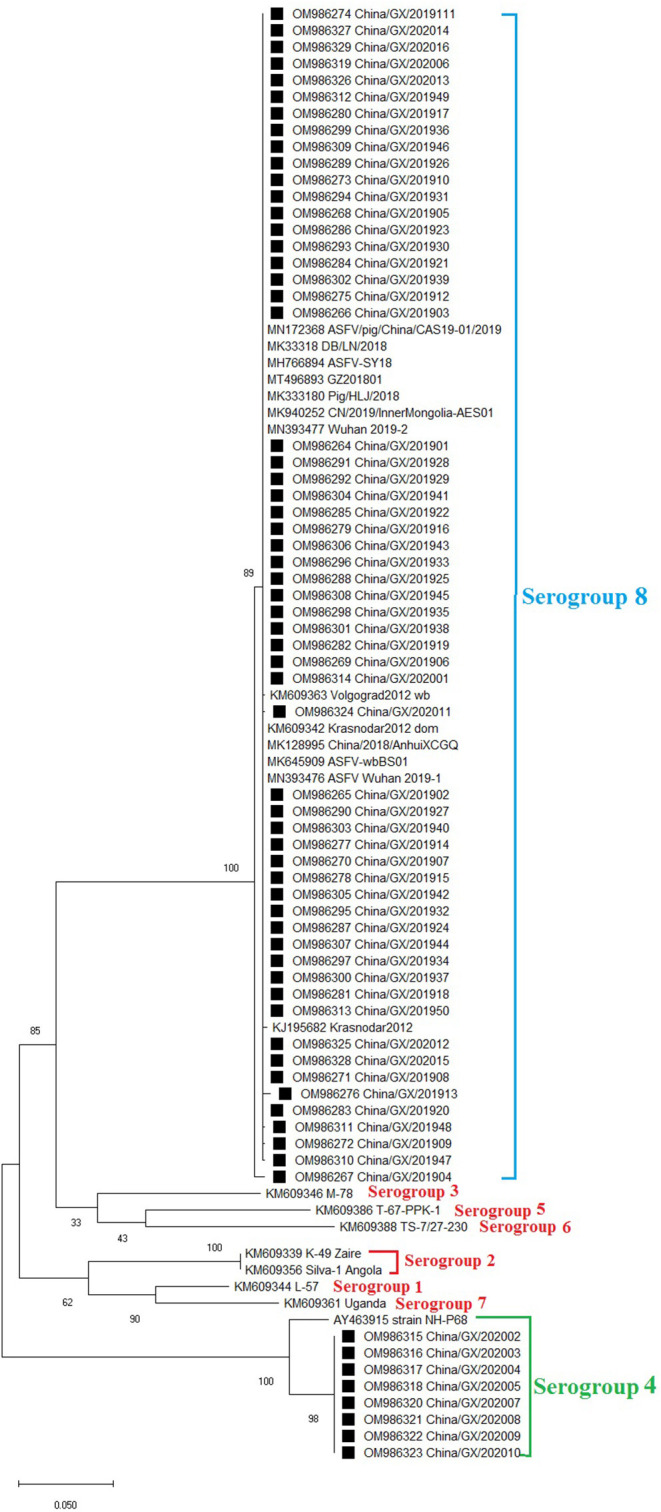
Phylogenetic tree based on nucleotide sequences of the *ER402R* (*CD2v*) gene. The evolutionary history was inferred by the maximum likelihood method based on the Tamura 3-parameter + G model. The phylogeny was inferred following 1,000 bootstrap replications, and node values showed percentage bootstrap support. The ASFV strains from Guangxi province were clustered into serogroups 4 and 8. The strains from this study were highlighted with black squares (■) before the names.

The *EP402R* (*CD2v*) gene was amplified, sequenced and analyzed. Phylogenetic analysis of *EP402R* (*CD2v*) nucleotide sequences revealed that all ASFV strains, including the 66 strains from this study (Table 2) and the reference strains downloaded from GenBank (Supplementary Table 3), could be clustered into eight serogroups. Of the 66 strains from this study, eight strains of genotype I belonged to serogroup four, and the other 58 strains of genotype II belonged to serogroup eight (Figure 3).

### Analysis of the CVR of the *B602L* Gene

**Figure 4:**
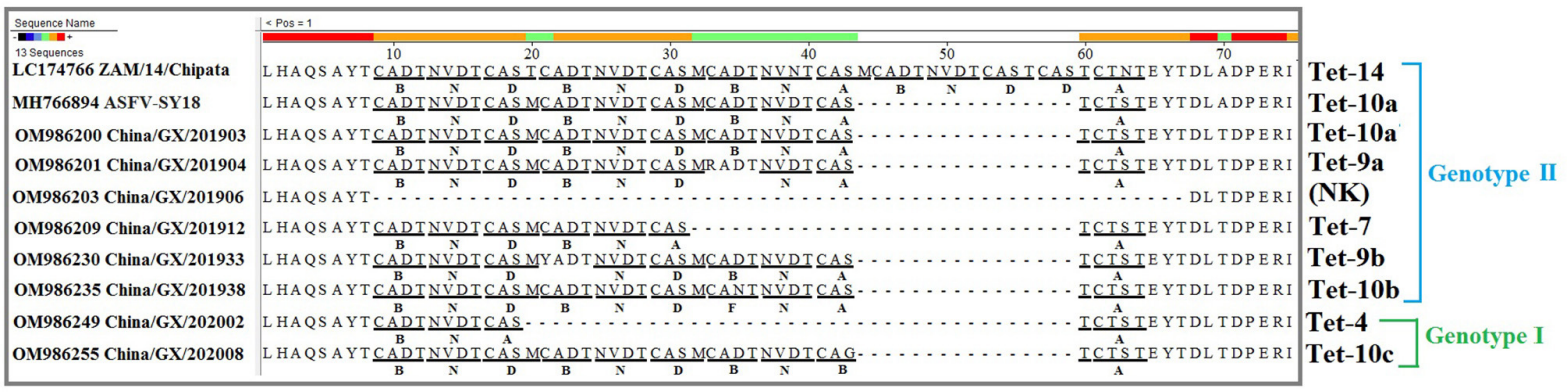
Amino acid sequence alignment of the tetrameric tandem repeat sequences (TRSs) of the central variable region (CVR) of the *B602L* gene. The ASFV strains from Guangxi province generated 8 different TRS variants. Only one representative strain of each variant was presented in this figure. Codes were as follows and as previously described: (CAST, CVST, CTST, CASI = A) (CADT, CADI, CTDT, CAGT, CVDT = B) (GAST, GANT = C) (CASM = D) (CANT, CAAT = F) (CTNT = G) (RAST = H) (GTDT = J) (CTSP = K) (YTNT = L) (NEDT = M) (NVDT, NVGT, NVDI = N) (NANI, NADI, NASI = O) (SAST = S) (NVNT = T) (NIDT, NTDT = U) (NAGT, NAST, NAVT, NADT, NANT = V) (SADT, SVDT = W) (NTDI = X).

The CVR of the *B602L* gene is a highly variable region and is often used for intragenotypic resolution of viruses belonging to the same *B646L* (*p72*) and *E183L* (*p54*) genotypes. Therefore, the CVR of the *B602L* gene of Guangxi ASFV strains was amplified, sequenced and analyzed. The results showed that the 66 strains from this study had different nucleotide lengths from 68 to 178 bp. The nucleotides were transformed into amino acids and analyzed. The sequencing analysis based on amino acids obtained eight different TRS variants from the circulating strains in Guangxi province (Table 2). Of the eight TRS variants, three variants (Tet-4, Tet-10a, and Tet-10c) belonged to genotype I, and six variants (NK, Tet-7, Tet-9a, Tet-9b, Tet-10a, and Tet-10b) belonged to genotype II, of which Tet-10a was found in both genotypes I and II. The tetrameric TRS of *BNDBNDBNAA* (Tet-10a) was the most dominant variant in this study. Sequence alignment revealed that four new tetrameric TRSs were found due to the mutation of a single amino acid (Figure 4), indicating the presence of single nucleotide polymorphism (SNP) in CVR variants. The similarity analysis of the tetrameric TRSs on NCBI database showed that, except for the sequence of *BNDBNDBNAA* (Tet-10a), the other seven TRS variants did not find 100% identical sequence. Furthermore, it was found that the CVR of *B602L* gene of the 66 strains was not affected by the region of source, the time of collection and the genotype of the strains.

### Analysis of the IGR Between the *I73R/I329L* Genes

**Figure 5:**
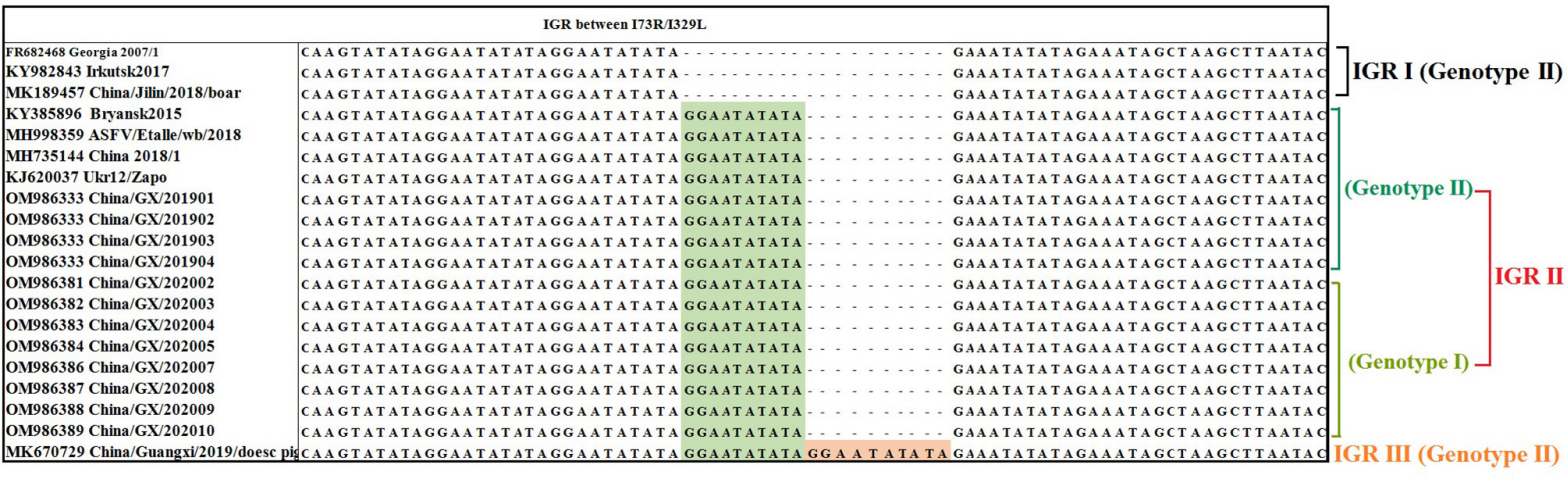
Nucleotide sequence alignment of the intergenic region (IGR) between *I73R/I329L* genes. The ASFV strains from Guangxi province belonged to IGR II with one insertion of 10 nucleotides (GGAATATATA) and IGR III with two insertions of 10 nucleotides (GGAATATATA). All the strains from this study belonged to IGR II and only partial strains were presented in this figure.

The IGR between the *I73R/I329L* genes was amplified, sequenced and characterized by the presence of TRSs. The results showed that all the 66 strains from Guangxi province in this study belonged to IGR II variant with an TRS insertion of the sequence GGAATATATA compared to the Georgia 2007/1 (GenBank accession no. FR682468.1), while one previously reported strain (China/Guangxi/2019/domestic pig, GenBank accession no. MK670729) from Guangxi province (36) belonged to IGR III variant with two TRS insertions, indicating that two types of variants (IGR II and IGR III variants) were co-circulating in Guangxi province during 2019–2020 outbreaks (Figure 5).

China/GX/202010 strain (GenBank accession no. OM986389) of genotype I and China/GX/201904 strain (GenBank accession no. OM986333) of genotype II from Guangxi province were selected as the representative strains for sequence similarity retrieval by BLAST software in the NCBI database (Table 3). The genotype II strain had 100% identity of the IGR between *I73R/I329L* genes with some strains from Eurasian countries, including Belarus, Ukraine, Russia, Belgium and Poland in Europe, and Mongolia, Vietnam, Indonesia and China in Asia. In addition, the IGR between *I73R/I329L* genes of the genotype I strain could not found a strain with 100% similarity in NCBI database, and the highest similar strains were the Portuguese strain OURT88/3 and the Spanish strain NHV with a similarity of 99.74%.

**Table 3:** Similarity analysis of the tandem repeat sequence (TRS) between *I73R/I329L* genes.

**Strain**	**Accession No**.	**Date**	**Origin**	**Fragment/bp**	**Similarity (%)**
**China/GX/202010 (genotype I)**					
China/GX/202010	OM986389	2020	China	385	–
NHV	NC044943	1968	Spain	385	99.74
OURT 88/3	NC044957	2004	Portugal	385	99.74
China/Guangxi/2019/domestic pig	MK670729	2019	China	356	91.37
Bel13/Grodno	KJ620043	2013	Belarus	367	90.72
Ukr12/Zapo	KJ620037	2012	Ukraine	366	90.72
VN/Pig/Hanoi/02	MW054561	2019	Vietnam	367	90.72
Indo/2020/Pig/West Java	MT851947	2020	Indonesia	363	90.65
**China/GX/201904 (genotype II)**					
China/GX/201904	OM986333	2019	China	366	–
VN/Pig/Hanoi/2019/01	MT332153	2020	Vietnam	366	100
Bel13/Grodno	KJ620043	2014	Belarus	366	100
Ukr12/Zapo	KJ620037	2014	Ukraine	366	100
VN/Pig/Hanoi/02	MW054561	2020	Vietnam	366	100
Indo/2020/Pig/West Java	MT851947	2020	Indonesia	363	100
Voronezh 2016	KY385893	2016	Russia	362	100
MNG/7/BU / 2019	MT852023	2019	Mongolia	357	100
CN2018/01	MH735144	2018	China	346	100
Belgium/2018/Etalle	MH998359	2018	Belgium	342	100
Tula06/2012	KP137625	2012	Russia	219	100
Pol20_36120_O52/20	MT951797	2020	Poland	217	100
China/Guangxi/2019/domestic pig	MK670729	2019	China	356	97.19
ASFV-wbBS01	MK238345	2019	China	341	97.15
China/Jilin/2018/boar	MK189457	2019	China	336	97.11
Arm07	KJ620028	2014	Armenia	356	97
VN/Pig/Hanoi/07	MW054562	2019	Vietnam	357	97
MAL/19/Karonga/4	MN755870	2019	Malawi	357	96.99
Tan_17_01	MK577991	2019	Tanzania	337	96.83
MAL/19/Karonga/2	MN755868	2019	Malawi	357	96.73
MAL/19/Karonga/1	MN755867	2019	Malawi	356	96.72
Irkutsk2017	KY982843	2017	Russia	355	96.69
4/Ol/02	KT718707	2017	Italy	345	90.23

*China/GX/202010 strain was used as the representative of genotype I, and China/GX/201904 strain was used as the representative of genotype II*.

## Discussion

ASF was first reported in China on August 3, 2018 (5), and then rapidly spread to most provinces in China within a short time (19). According to a report from the Ministry of Agriculture and Rural Affairs of China, by August 2019, ASF had caused a loss of 190.9 million pigs, including 29.4 million sows with a 40.5% decrease in China (37), showing that ASF had caused huge losses to the pig industry within a very short time. It has been reported that genotypes I and II of ASFV were found to be simultaneously epidemic in the pig herds in China (20, 21), and the wild-type ASFV strains with different virulence and the naturally gene-deleted ASFV strains with decreased virulence were identified in several provinces in China (22, 23), indicating that many different ASFV strains with high genetic diversity were circulating in China. Therefore, the molecular characterization of ASFV strains circulating in China, which has not been reported in detail until now, is necessary to further study.

The tissue samples from dead pigs collected from 86 pig farms during 2019–2020 in Guangxi province, southern China, were tested for ASFV, and a high positive rate of 57.14% (192/336) was found, indicating that ASF occurred seriously in these pig farms. To further analyze the molecular characterization of the circulating ASFVs, 66 positive samples were randomly selected for gene sequencing and analysis. These samples were also tested by a commercial ASFV qPCR detection kit (Lijian, China), and 8 ASFV strains lacked *MGF505-2R* gene. These situations were further confirmed by the sequence alignment. Recently, there were reports on ASFV strains with different levels of virulence causing a broad range of clinical symptoms in susceptible animals. Per-acute and acute ASFV infections might lead to mortality rate up to 100% in naïve domestic pig populations (5, 38, 39), while pigs infected with low virulence strains could develop resistance to ASFV leading to an increased number of chronic or subclinical infections (20, 22, 40, 41). Some researchers have reported that the gene-deleted ASFV strains showed decreased virulence and low pathogenicity, such as the non-pathogenic strain OURT88/3 in Portugal lacking the *MGF505-2R* gene (42), and the attenuated strain BA71 in Spain losing its *EP402R* (*CD2v*) gene (43). The naturally gene-deleted ASFV strains with decreased virulence has been reported in several provinces in eastern China (22, 23), and the man-made gene-deleted strains with decreased virulence have also been reported in China (44). Of the 66 strains in this study, eight strains lacked *MGF505-2R* gene. Therefore, the wild-type and the gene-deleted ASFV strains were simultaneously circulating during 2019–2020 in Guangxi province, southern China, and their harm to the pig industry needs to be further evaluated.

ASF was first reported in Kenya in the 1920s, introduced into Georgia in 2007 and has subsequently spread to Russia, eastern Europe, Asia and Latin America with devastating socioeconomic consequences (3, 8, 9, 45, 46). To date, two of the 24 currently described ASFV genotypes defined by the *B646L* (*p72*) gene, namely genotypes I and II, have been reported outside Africa, with genotype II being responsible for the ongoing ASF pandemic (13, 14). In this study, the positive clinical samples, collected during 2019–2020 outbreaks from Guangxi province, were randomly selected for gene sequence analysis. The *B646L* (*p72*) and *E183L* (*p54*) gene sequences were used to determine the genotype of the viruses by phylogenetic analysis, while the *EP402R* (*CD2v*) gene sequence was analyzed to determine the serogroup. Phylogenetic analysis of the *B646L* (*p72*) gene revealed that all 66 strains from Guangxi province belonged to genotypes I (8/66) and II (58/66), and phylogenetic analysis of the *E183L* (*p54*) gene revealed that all 66 strains from Guangxi province belonged to genotypes Ia (8/66) and IIa (58/66), indicating that the genotyping by the *B646L* (*p72*) and *E183L* (*p54*) nucleotide sequences generated similar genotypes for the circulating ASFV strains in Guangxi province. Phylogenetic analysis of the *EP402R* (*CD2v*) gene revealed that the strains from Guangxi province could be clustered into two serogroups, serogroups 4 (8/66) and 8 (58/66). Traditionally, genotyping of ASFV strains depended on the phylogenetic analysis of the *B646L* (*p72*) and *E183L* (*p54*) gene sequences, and the analysis of the *E183L* (*p54*) gene sequences could improve the identification of each genotype (30, 32, 33, 47, 48). However, some scholars proposed that the *B646L* (*p72*) gene could not accurately define the serogroup of ASFV, distinguish viruses with different virulence, or predict the effectiveness of specific ASFV vaccines, so they advocated to determine the serogroup of ASFVs based on the phylogenetic analysis of the *EP402R* (*CD2v*) gene (34, 49, 50). Therefore, it is recommended that genotyping should be combined with serotyping for epidemiological investigation of ASFV.

Many reports have used the genetic characteristics of the CVR in the *B602L* gene for genotyping and subgrouping closely related ASFV strains (25, 30–35, 48–52). In this study, the ASFV strains circulating in Guangxi province from 2019 to 2020 presented a multi-complex profile with a high degree of heterogeneity that could be clustered into 8 different TRS variants, and three variants belonged to genotype I and six variants belonged to genotype II (Table 2). To better track the source of these strains, the similarity analysis between these 8 TRS variants and the strains from GenBank database showed that only the TRS variant *BNDBNDBNAA* had 100% similar sequences, and the other seven variants could not find a completely consistent sequence. In addition, the situation that the TRSs within CVR of some genotype I strains was identical to those of the genotype II strains was also reported previously (50). The results demonstrated that the CVR in the *B602L* gene is a hypervariable genetic marker for high-resolution discrimination of viruses that are identical based on their *B646L* (*p72*) and *E183L* (*p54*) genotypes.

The CVR of the *B602L* gene has been widely used to distinguish closely related ASFVs, but the relatively low CVR genetic variability necessitates further research on alternative and more informative gene regions, so the sequence analysis of the IGR between *I73R/I329L* genes is used to determine the relationship within the relevant genotypes and origin of circulating ASFV strains (28, 29, 32, 34, 36, 49, 52–55). According to the results in this study, the IGR II and IGR III variants were simultaneously circulating in Guangxi province (Figure 5). Furthermore, the circulating genotype II strains in Guangxi province had 100% identity with some strains from Eurasian countries (Table 3). In addition, the IGR between *I73R/I329L* genes of the circulating genotype I strains in Guangxi province did not find a 100% similar sequence on NCBI database, and the most similar strain was the Portuguese OURT88/3 strain and Spanish NHV strain with 99.74% similarity. Therefore, Chinese ASFV strains, including the strains form Guangxi province, might be derived from Europe, and more genome sequences of ASFV strains circulating in pig herds will help precisely trace the origin of ASFV strains in China.

In conclusion, this study first reported that ASFV strains of two genotypes (genotypes I and II based on the *B646L* (*p72*) and *E183L* (*p54*) genes) and two serogroups (serogroups 4 and 8 based on the *EP402R* (*CD2v*) gene) were simultaneously circulating during 2019–2020 outbreaks in Guangxi province, southern China. There existed wild-type and naturally gene-deleted ASFV strains in the field. These strains generated eight different CVR variants within the *B602L* gene and belonged to IGR II and III variants between *I73R/I329L* genes. To our knowledge, this is the first detailed report on the molecular characterization of ASFV strains circulating in southern China, and serogroup 4 ASFV strains in China.

## Data Availability Statement

The datasets presented in this study can be found in online repositories. The names of the repository/repositories and accession number(s) can be found in the article/Supplementary Material.

## Author Contributions

KS contributed to study design, laboratory supervision, and manuscript writing and editing. HL contributed to experiments, data analysis, and manuscript drafting. YY, FL, and SF contributed to sample collection and data analysis. HS contributed to study design and manuscript editing. All authors have read and approved the submitted manuscript.

## Funding

This study was supported by the Key Research and Development Program (AB21238003) and the Science and Technology Major Project (AA17204057) of Guangxi Science and Technology Bureau, China, and the Agricultural Science and Technology Program (Z201954, Z202031) of Guangxi Agricultural and Rural Bureau, China.

## Conflict of Interest

The authors declare that the research was conducted in the absence of any commercial or financial relationships that could be construed as a potential conflict of interest.

## Publisher's Note

All claims expressed in this article are solely those of the authors and do not necessarily represent those of their affiliated organizations, or those of the publisher, the editors and the reviewers. Any product that may be evaluated in this article, or claim that may be made by its manufacturer, is not guaranteed or endorsed by the publisher.
