# Supplementary material for: Molecular Characterization of African Swine Fever Virus From 2019-2020 Outbreaks in Guangxi Province, Southern China
Source: Front Vet Sci. 2022 Jun 15;9:912224. doi: 10.3389/fvets.2022.912224 (PMC9240437; doi:10.3389/fvets.2022.912224)
Supplement: Supplementary file 1 [file Table_1.DOCX]

**Supplementary**

TABLE S1. The reference sequences of ASFV *p72* gene used in this study

TABLE S2. The reference sequences of ASFV *p54* gene used in this study

TABLE S3. The reference sequences of ASFV *CD2v* gene used in this study

**TABLE S1.** **The reference sequences of ASFV *p72* gene used in this study**

| **Strain** | **Accession No.** | **Origin** | **Date** | ***p72*** | **Reference** |
| --- | --- | --- | --- | --- | --- |
| MAD/1/98 | AF270706 | Mozambia | 2000 | II | Bastos et al. (2003) |
| SPEC265 | AF270710 | Mozambia | 2000 | VI | Bastos et al. (2003) |
| MOZ/94/1 | AF270711 | Mozambia | 2000 | VI | Bastos et al. (2003) |
| Tengani | AF301541 | Malawi | 2000 | V | Bastos et al. (2003) |
| CAM/4/85 | AF301545 | Cameroon | 2000 | I | Bastos et al. (2003) |
| BUR/1/84 | AF449463 | Uganda | 2001 | X | Bastos et al. (2003) |
| ZAR85 | AF449465 | Spain | 2001 | I | Bastos et al. (2003) |
| UGA/1/95 | AF449475 | Uganda | 2001 | IX | Bastos et al. (2003) |
| UGA/3/95 | AF449476 | Uganda | 2001 | X | Bastos et al. (2003) |
| RSA/1/99/W | AF449477 | South Africa | 2001 | IV | Bastos et al. (2003) |
| NIG-2 | AF504884 | Nigeria | 2002 | I | Bastos et al. (2003) |
| BOT/1/99 | AF504886 | Botswana | 2002 | III | Bastos et al. (2003) |
| GHA/1/00 | AF504888 | Ghana | 2002 | I | Bastos et al. (2003) |
| MOZ-60/98 | AY274455 | Mozambia | 2003 | II | Bastos et al. (2003) |
| KAB/62 | AY351522 | Zambia | 2003 | XI | Lubisi et al. (2005) |
| SUM/1411 | AY351542 | Zambia | 2003 | XIII | Lubisi et al. (2005) |
| MZI/921 | AY351543 | Malawi | 2003 | XII | Lubisi et al. (2005) |
| THY/901 | AY351545 | Malawi | 2003 | VIII | Lubisi et al. (2005) |
| MWHOG/3 | AY351549 | Malawi | 2003 | X | Lubisi et al. (2005) |
| NYA/12 | AY351555 | Zambia | 2003 | XIV | Lubisi et al. (2005) |
| LUS93/1 | AY351563 | Zambia | 2003 | II | Lubisi et al. (2005) |
| TAN/2003/1 | AY494550 | Tanzania | 2003 | XVI | Lubisi et al. (2005) |
| TAN/1/01 | AY494552 | Tanzania | 2003 | XV | Lubisi et al. (2005) |
| SPEC/154 | DQ250113 | Botswana | 2005 | VII | Boshoff et al. (2007) |
| SPEC/245 | DQ250117 | South Africa | 2005 | XXII | Boshoff et al. (2007) |
| ZIM/92/1 | DQ250119 | Zimbabwe | 2005 | XVII | Boshoff et al. (2007) |
| SPEC/260 | DQ250121 | South Africa | 2005 | VII | Boshoff et al. (2007) |
| NAM/1/95 | DQ250122 | Namibia | 2005 | XVIII | Boshoff et al. (2007) |
| RSA/1/95 | DQ250123 | South Africa | 2005 | XX | Boshoff et al. (2007) |
| RSA/1/96 | DQ250125 | South Africa | 2005 | XXI | Boshoff et al. (2007) |
| RSA/2/96 | DQ250126 | South Africa | 2005 | XIX | Boshoff et al. (2007) |
| Ug03H.1 | FJ154428 | Uganda | 2008 | IX | Gallardo et al. (2009) |
| Ken06.B3 | FJ154436 | Spain | 2008 | IX | Gallardo et al. (2009) |
| Kat67 | FJ174377 | Congo | 2008 | I | Gallardo et al. (2009) |
| Ang72 | FJ174378 | Angola | 2008 | I | Gallardo et al. (2009) |
| Nig01 | FJ174382 | Nigeria | 2008 | I | Gallardo et al. (2009) |
| Ken05/Tk5 | HM745257 | Kenya | 2010 | X | Gallardo et al. (2009) |
| Tan 2011/01 | JX310038 | Tanzania | 2011 | II | Unpublished |
| KEN 2001/2 | JX524215 | Kenya | 2001 | IX | Unpublished |
| TAN/13/Moshi | KF706360 | Tanzania | 2013 | X | Misinzo et al. (2014) |
| ETH/3 | KT795360 | Ethiopia | 2011 | XXIII | Achenbach et al. (2016) |
| MOZ_16/2006 | KY353995 | Mozambia | 2006 | XXIV | Quembo et al. (2017) |
| ASFV-SY18 | MH766894 | China | 2018 | II | Unpublished |
| China/2018/AnhuiXCGQ | MK128995 | China | 2018 | II | Bao et al. (2019) |
| Pig/HLJ/2018 | MK333180 | China | 2018 | II | Wen et al. (2019) |
| DB/LN/2018 | MK333181 | China | 2018 | II | Wen et al. (2019) |
| ASFV-wbBS01 | MK645909 | China | 2018 | II | Unpublished |
| CN/2019/InnerMongolia-AES01 | MK940252 | China | 2019 | II | Unpublished |
| ASFV/pig/China/CAS19-01/2019 | MN172368 | China | 2019 | II | Jia et al. (2019) |
| ASFV Wuhan 2019-1 | MN393476 | China | 2019 | II | Xiong et al. (2020) |
| ASFV Wuhan 2019-2 | MN393477 | China | 2019 | II | Xiong et al. (2020) |
| GZ201801 | MT496893 | China | 2018 | II | Unpublished |

**TABLE S2.**  **The reference sequences of ASFV *p54* gene used in this study**

| **Strain** | **Accession No.** | **Origin** | **Date** | ***p54*** | **Reference** |
| --- | --- | --- | --- | --- | --- |
| ASFV-SY18 | MH766894 | China | 2018 | II | Unpublished |
| China/2018/AnhuiXCGQ | MK128995 | China | 2018 | II | Bao et al. (2019) |
| Pig/HLJ/2018 | MK333180 | China | 2018 | II | Wen et al. (2019) |
| DB/LN/2018 | MK333181 | China | 2018 | II | Wen et al. (2019) |
| ASFV-wbBS01 | MK645909 | China | 2018 | II | Unpublished |
| CN/2019/InnerMongolia-AES01 | MK940252 | China | 2019 | II | Unpublished |
| ASFV/pig/China/CAS19-01/2019 | MN172368 | China | 2019 | II | Jia et al. (2019) |
| ASFV Wuhan 2019-1 | MN393476 | China | 2019 | II | Xiong et al. (2020) |
| ASFV Wuhan 2019-2 | MN393477 | China | 2019 | II | Xiong et al. (2020) |
| GZ201801 | MT496893 | China | 2018 | II | Unpublished |
| ZAM/14/Chipata | LC174760 | Zambia | 2014 | II | Simulundu et al. (2017) |
| ETH/1 | KT795366 | Ethiopia | 2016 | XXIII | Achenbach et al. (2016) |
| SUM/141 | EU874357 | South Africa | 2008 | XIII | Unpublished |
| MwLil20/1 | FJ174425 | Malawi | 1983 | VIIIa | Gallardo et al. (2009) |
| KAB/62 | EU874331 | South Africa | 2008 | XI | Unpublished |
| DRC/21/07/22 | KX121483 | Congo | 2007 | XIV | Unpublished |
| Moz64 | FJ174422 | Mozambique | 1964 | Vb | Gallardo et al. (2009) |
| ZAM/13/Lusaka | LC174764 | Zambia | 2013 | Id | Simulundu et al. (2017) |
| Lisbon 60 | X84889 | Portugal | 1995 | Ic | Unpublished |
| IC96 | FJ174429 | Cote d'Ivoire | 1996 | Ib | Gallardo et al. (2009) |
| Co62 | FJ238536 | Haiti | 1981 | Ia | Gallardo et al. (2009) |
| Pig 951 | KF015872 | South Africa | 1979 | VIIIa | Unpublished |
| MAL 2011/1 | KF015982 | Malawi | 2011 | IIc | Unpublished |
| NAM 2011/1 | KF015975 | Namibia | 2011 | Ig | Unpublished |
| LIV 10/11 | KF015932 | Zambia | 1983 | Ie | Unpublished |
| ZAM 2001/5 | KF015917 | Zambia | 2001 | If | Unpublished |
| Ken06.B2 | FJ174442 | Kenya | 2009 | Vb | Gallardo et al. (2009) |
| Ug64 | FJ174430 | Uganda | 1964 | Xa | Gallardo et al. (2009) |
| Lillie 148 | X84888 | South Africa | 1979 | XXb | Unpublished |
| Ug03H.1 | FJ174431 | Uganda | 2003 | IX | Gallardo et al. (2009) |

**TABLE S3.**  **The reference sequences of ASFV *CD2v* gene used in this study**

| **Strain** | **Accession No.** | **Origin** | **Date** | ***CD2v*** | **Reference** |
| --- | --- | --- | --- | --- | --- |
| NH-P68 | AY463915 | Portuguesa | 2003 | IV | Rowlands et al. (2009) |
| Krasnoda2012 | KJ195682 | Russia | 2012 | VIII | Unpublished |
| Volgograd_2012/wb | KM609363 | Russia | 2012 | VIII | Malogolovkin et al. (2015) |
| Krasnodar_2012/dom | KM609342 | Russia | 2012 | VIII | Malogolovkin et al. (2015) |
| M-78 | KM609346 | Mozambique | 1978 | III | Malogolovkin et al. (2015) |
| K-49 | KM609339 | Zaire | 1949 | II | Malogolovkin et al. (2015) |
| Silva-1 | KM609356 | Angola | 1982 | II | Malogolovkin et al. 2015) |
| Uganda | KM609361 | Uganda | 2014 | VII | Malogolovkin et al. (2015) |
| T-67-PPK-1 | KM609386 | Tanzania | 2014 | V | Malogolovkin et al. (2015) |
| L-57 | KM609344 | Portuguesa | 1957 | I | Malogolovkin et al. (2015) |
| TS-7/27-230 | KM609388 | Tanzania | 2014 | VI | Malogolovkin et al. (2015) |
| ASFV-SY18 | MH766894 | China | 2018 | II | Unpublished |
| China/2018/AnhuiXCGQ | MK128995 | China | 2018 | II | Bao et al. (2019) |
| Pig/HLJ/2018 | MK333180 | China | 2018 | II | Wen et al. (2019) |
| DB/LN/2018 | MK333181 | China | 2018 | II | Wen et al. (2019) |
| ASFV-wbBS01 | MK645909 | China | 2018 | II | Unpublished |
| CN/2019/InnerMongolia-AES01 | MK940252 | China | 2019 | II | Unpublished |
| ASFV/pig/China/CAS19-01/2019 | MN172368 | China | 2019 | II | Jia et al. (2019) |
| ASFV Wuhan 2019-1 | MN393476 | China | 2019 | II | Xiong et al. (2020) |
| ASFV Wuhan 2019-2 | MN393477 | China | 2019 | II | Xiong et al. (2020) |
| GZ201801 | MT496893 | China | 2018 | II | Unpublished |
